# Nucleocytoplasmic distribution of S6K1 depends on the density and motility of MCF-7 cells *in vitro*

**DOI:** 10.12688/f1000research.15447.2

**Published:** 2018-12-17

**Authors:** Viktoriia Kosach, Kateryna Shkarina, Anastasiia Kravchenko, Yuliia Tereshchenko, Evelina Kovalchuk, Larysa Skoroda, Mykhailo Krotevych, Antonina Khoruzhenko

**Affiliations:** 1 Institute of Molecular Biology and Genetics, National Academy of Sciences of Ukraine, Kyiv, 03143, Ukraine; 2 Educational and Scientific Center , Taras Shevchenko National University of Kyiv, Kyiv, 03022, Ukraine; 3 National Cancer Institute, Kyiv, 03022, Ukraine

**Keywords:** S6K1, subcellular localization, mTOR/S6K1 signaling pathway, breast cancer, TBR2, Eomesodermin, MCF-7 cell line

## Abstract

**Background:** The ribosomal protein S6 kinase 1 (S6K1) is one of the main components of the mTOR/S6K signal transduction pathway, which controls cellular metabolism, autophagy, growth, and proliferation. Overexpression of S6K1 was detected in tumors of different origin including breast cancer, and correlated with the worse disease outcome. In addition, significant accumulation of S6K1 was found in the nuclei of breast carcinoma cells suggesting the implication of kinase nuclear substrates in tumor progression. However, this aspect of S6K1 functioning is still poorly understood. The main aim of the present work was to study the subcellular localization of S6K1 in breast cancer cells with the focus on cell migration.

**Methods:** Multicellular spheroids of MCF-7 cells were generated using agarose-coated Petri dishes. Cell migration was induced by spheroids seeding onto adhesive growth surface and subsequent cultivation for 24 to 72 hours. The subcellular localization of S6K1 was studied in human normal breast and cancer tissue samples, 2D and 3D MCF-7 cell cultures using immunofluorescence analysis and confocal microscopy.

**Results:** Analysis of histological sections of human breast tissue samples revealed predominantly nuclear localization of S6K1 in breast malignant cells and its mainly cytoplasmic localization in conditionally normal cells. *In vitro* studies of MCF-7 cells demonstrated that the subcellular localization of S6K1 depends on the cell density in the monolayer culture. S6K1 relocalization from the cytoplasm into the nucleus was detected in MCF-7 cells migrating from multicellular spheroids onto growth surface. Immunofluorescence analysis of S6K1 and immunocoprecipitation assay revealed the colocalization and interaction between S6K1 and transcription factor TBR2 (T-box brain protein 2) in MCF-7 cells.

**Conclusions:** Subcellular localization of S6K1 depends on the density and locomotor activity of the MCF-7 cells.

## Introduction

Ribosomal protein S6 kinase 1 (S6K1) belongs to the AGC family of serine/threonine protein kinases ( Ruvinsky & Meyuhas, 2006). It is involved in the regulation of crucial physiological processes, such as protein synthesis, ribosomal biogenesis, the G1/S-phase cell cycle transition, mRNA splicing, differentiation of specific cell types, and apoptosis. The large number of intracellular targets makes S6K1 a key regulator of cell size, growth, and proliferation ( Magnuson *et al*., 2012). S6K1 activity is controlled by the PI3K/Akt/mTOR signaling pathway, which has been shown to be dysregulated in diverse human pathologies, including diabetes, obesity, neurodegenerative disorders, and cancer ( Tavares *et al*., 2015). Overexpression of S6K1 was found in several tumor types, including breast cancer, and was associated with the worse disease outcome for the patients ( Bostner *et al*., 2015).

In mammalian cells, S6K1 is encoded by *RPS6KB1* gene located at the chromosome 17. Several isoforms of the S6K1 protein are known: the 85kDa S6K1 and the 70kDa S6K1 (p85S6K1 and p70S6K1 respectively), which originate from alternative translation initiation sites, and hypothetical p60S6K1, which is also suggested to be a product of alternate mRNA translation ( Kim *et al*., 2009). Recently, the new 31kDa isoform of S6K1 (p31S6K1) encoded by mRNA splice variant was also identified. Although it has been shown that p31S6K1 protein lacks the catalytic activity of the kinase domain, it still possesses oncogenic properties ( Ben-Hur *et al*., 2013; Song & Richard, 2015). The longer isoform p85S6K1 has an additional 23 amino acid extension at the N-terminus of the molecule functioning as a nuclear localization signal. In early studies p85S6K1 was described as a predominantly nuclearly localized kinase. However, recent studies based on nuclear-cytoplasmic fractionation revealed its presence in the cytoplasm of the breast cancer cells and primary human fibroblasts ( Kim *et al*., 2009; Rosner & Hengstschläger, 2011). The most abundant isoform of S6 kinase, p70S6K1, was thought to localize predominantly in the cytoplasm, however treatment of the cells with leptomycin B (the nuclear export inhibitor) has been shown to cause its accumulation in the nucleus, leading to the conclusion that p70S6K1 may shuttle between the cytoplasm and nucleus of the cell ( Panasyuk *et al*., 2006). To date, there is still very little evidence about the subcellular localization of p31S6K1. It is proposed to be present in the nuclei of human normal fibroblasts ( Rosner & Hengstschläger, 2011). Overall, S6K1 subcellular localization data have been based predominantly on subcellular fractionation assay or immunocytochemical analysis of recombinantly expressed proteins. However, information about the nucleocytoplasmic distribution of the endogenous S6K1 is still limited, and mechanisms of its regulation remain elusive.

Recent studies suggest that S6K1 subcellular localization and activation may also depend on the physiological status of different tissues. Immunohistochemical analysis of malignant breast tumors revealed prominent S6K1 accumulation in the nuclei of carcinoma cells ( Filonenko, 2013; Filonenko *et al*., 2004; Lyzogubov *et al*., 2005). In other studies, it was shown that nuclear accumulation of S6K1 correlated with the reduced tamoxifen effect in breast cancer patients, while cytoplasmic localization of S6K1 was associated with better prognosis for the patients ( Bostner *et al*., 2015).

Migration of the cancer cells is an important stage of cancer progression, leading the tumor invasion and formation of distant metastases. The recent data suggest that S6K1 may be involved in the regulation of the motility of normal and malignant cells, as knockdown of p70S6K1 or inhibition of S6K1 kinase activity caused a significant decrease in the migration speed of the prostate, breast, and ovarian cancer cells *in vitro* ( Amaral *et al*., 2016; Ip *et al*., 2011). Moreover, activation of p70S6K1 in human ovarian carcinoma cells in response to stimulation by hepatocyte growth factor (HGF) also led to increased expression of matrix metalloproteinase 9 (MMP9) and higher migration rate of these cells ( Zhou & Wong, 2006). It was shown that active p70S6K1 could also induce activation of Cdc42, Rac1, and PAK1 – the known regulators of cell migration through actin cytoskeleton remodelling ( Aslan *et al*., 2011; Liu *et al*., 2010). Besides, S6K1 was also found to colocalize with the actin arches at the leading edge of moving mesothelioma cells. Treatment with rapamycin (specific mTOR inhibitor) reduced the formation of actin arches even when cells were stimulated with epithelial growth factor (EGF) ( Berven *et al*., 2004; Liu *et al*., 2008). However, the link between subcellular localization of S6K1 and its functions in migrating cancer cells is not fully yet understood.

In the present research, we focused on the study of subcellular localization of endogenous S6K1 in breast tumor and normal tissue, and in breast adenocarcinoma MCF-7 cells in monolayer culture, 3D multicellular spheroids, and in the course of induced cancer cell migration. We found that nucleocytoplasmic distribution of S6K1 depends greatly on the density of the monolayer culture, and is different between the cells in 3D vs 2D culture conditions. Moreover, we found that S6K1 is relocalized to the nucleus during migration of MCF-7 cells from multicellular spheroids onto growth surface. In addition, we analyzed the possible interaction of S6K1 with a number of transcription factors, involved in the regulation of cell motility. For the first time, we described the interaction of S6K1 and TBR2 (T-box brain protein 2) in breast cancer cell line MCF-7. Together, these data suggest that during cell migration S6K1 interacts with the transcription factors in the cell nucleus, leading to the possibility of its transcriptional regulation of the genes that are involved in the control of cellular locomotor activity.

## Methods

### Cell culture

Human breast adenocarcinoma cell line MCF-7 was obtained from Bank of Cell Lines of the R. E. Kavetsky Institute of Experimental Pathology, Oncology and Radiobiology, NASU (Ukraine). The cells were cultivated in DMEM culture medium (Gibco, USA) supplemented with 10% fetal calf serum (FCS, HyClone, USA), 4 mM glutamine, 50 units/ml penicillin, 50 µg/ml streptomycin at 37°C in presence of 5% CO _2_. The medium was exchanged every third day. For immunofluorescence analysis cells were seeded onto sterile glass coverslips 48 hours before the experiments.

To form multicellular spheroids, MCF-7 cells were trypsinized, and 1×10 ^6^ cells were seeded into 100 mm Petri dishes, that were previously coated with 1% agarose (Sigma-Aldrich, A9045), and left to form the multicellular aggregates for the additional 72 h.

For the induction of spheroid-to-monolayer transition and cell migration, multicellular spheroids were transferred onto growth surface (glass coverslip) and further cultured for 24 to 72 h. Then outspreaded spheroids were fixed and used for immunofluorescence analysis.

Cellular and spheroid morphology was also evaluated using transmitted light microscopy (CETI Versus inverted microscope, CETI, Belgium, and Leica DM 1000, Leica Microsystems, Germany).

### Immunofluorescence analysis

MCF-7 cells were fixed with 10% formalin for 15 min at room temperature (RT). After this, the cells were permeabilized with 0.2% Triton X-100 in PBS for 10 min. To reduce autofluorescence, the samples were incubated with 10 mM cupric sulphate and 50 mM ammonium acetate, pH 5.0 for 30 min at RT. Non-specific binding was blocked through the incubation with 10% FCS in PBS for 30 min at 37°C in a humidified chamber.

S6K1 subcellular localization was revealed using anti-S6K1-C-terminus rabbit polyclonal antibodies (generated and evaluated earlier ( Savinska *et al*., 2001; if you are interested in obtaining this antibody, please contact the corresponding author)) at 1:100, and anti-phospho-S6K1 (T389) rabbit polyclonal antibodies at 1:20 (Cell Signaling Technology Cat# 9205, RRID:AB_330944). The secondary Fluorescein (FITC)-AffiniPure Goat Anti-Rabbit IgG (H+L) antibody 1:400 (Jackson ImmunoResearch Labs Cat# 111-095-003, RRID:AB_2337972) were applied for 45 min at 37°C in a humidified chamber.

Double immunofluorescence analysis was performed by addition of the primary antibody mix: anti-S6K1-C-terminal mouse monoclonal antibodies (generated earlier ( Pogrebnoy *et al*., 1999); if you are interested in obtaining this antibody, please contact the corresponding author) at 1:100 + anti-TBR2 rabbit polyclonal antibodies at 1:100 (Abcam Cat# ab23345, RRID:AB_778267) overnight at +4°C in a humidifyied chamber. The secondary Fluorescein (FITC)-AffiniPure Donkey Anti Mouse IgG (H+L) antibody (Jackson ImmunoResearch Labs Cat# 715-095-150, RRID:AB_2340792) at 1:400, and Rhodamine (TRITC)-AffiniPure Donkey Anti-Rabbit IgG (H+L) antibody (Jackson ImmunoResearch Labs Cat# 711-025-152, RRID:AB_2340588) at 1:400 were applied for 45 min at 37°C in a humidifying chamber. Samples were embedded into Mowiol medium (Sigma-Aldrich, USA) containing 2.5% DABCO (Sigma-Aldrich), 0.5 % DAPI (Sigma-Aldrich).

Microscopy image acquisition was performed using Leica DM 1000 epifluorescent microscope and Zeiss LSM 510 META point scanning confocal microscope (Carl Zeiss Microscopy GmbH, Germany). Fluorescence images were analyzed using Fiji/ImageJ v1.52b ( Fiji, RRID:SCR_002285; Schindelin *et al*., 2012). Figures were generated with the FigureJ plugin ( Mutterer & Zinck, 2013) in Fiji/ImageJ v1.52b.

For quantitative characterization of colocalization Pearson coefficient and Manders coefficients (M1 and M2) analysis was performed on the background-subtracted images using JACoP plugin ( Bolte & Cordelières, 2006) in Fiji/ImageJ v1.52b. Pearson coefficient (Rr) and Manders coefficients (M1 and M2) were expressed as mean value +/-SD, the experiments were performed in duplicates. To validate and describe the obtained degree of colocalization pre-defined image sets from Colocalization Benchmark Source were used. Obtained values of the colocalization coefficients were used to find the closest benchmark.

### Immunohistochemical analysis

Histological samples of human mixed ductal/lobular carcinoma of the breast and surrounding conditionally normal tissue were obtained from 10 patients within the framework of the cooperation agreement between the National Cancer Institute and the Institute of Molecular Biology and Genetics of the National Academy of Sciences of Ukraine. This study has been approved by the Committee on Biological & Medical Ethics of the National Cancer Institute of Ukraine (approval number - № 67, 25.03.2015). Written informed consent was obtained from all patients for the use of their tissues in research.

Sections of human breast cancer and surrounding tissues or multicellular spheroids were deparaffinized in xylene and rehydrated in a series of graded alcohol solutions. For the antigen retrieval, slides were placed in citrate buffer (10 mM citric acid, pH 6.0) and subsequently boiled two times for 5–7 min. Then, sections were treated with 0.2% Triton X-100 for 10min. Endogenous peroxidase was quenched by incubation of the samples with the 3% H _2_O _2_ in PBS for 30 min. After blocking of non-specific staining with 10% FCS in PBS, sections were incubated with anti-S6K1-C-terminal rabbit polyclonal antibodies (1:100) overnight at +4°C, and next with the peroxidase-conjugated secondary antibodies (1:100; Promega Cat# W4011, RRID:AB_430833) for 1 hour at 37°C. The reaction was developed with 3,3’diaminobenzidine (Sigma-Aldrich) solution.

### Bioinformatic analysis

Prediction of potential TBR2 phosphorylation sites by S6K1 was performed using Group-based Prediction System v2.1 ( Xue *et al*., 2011; GPS, RRID:SCR_016374). The sequence of human TBR2 was obtained from the National Center for Biotechnology Information, NCBI Reference Sequence: NP_001265111.1.

### Immunoprecipitation and immunoblot analysis

Anti-S6K1 mouse monoclonal antibodies ( Pogrebnoy *et al*., 1999) were immobilized on protein A/G PLUS Agarose beads (Santa Cruz Biotechnology) overnight at +4 C.

MCF-7 cells were washed with ice-cold PBS and extracted with lysis buffer, containing 20 mM Tris-HCl (pH 7.5), 150 mM NaCl, 1% Triton X-100, 5 mM EDTA, 50 mM sodium fluoride, 5 mM β-glycerophosphate,10 mM sodium pyrophosphate, 1 mM sodium orthovanadate and a mixture of protease inhibitors (Roche Molecular Diagnostics, France). Cell lysates were centrifuged at 13 000 rpm for 20 min at 4°C. Endogenous S6K1 was precipitated by adding 1000µg of total cell lysates to the immobilized antibodies and incubating overnight at 4°C. Immune complexes were washed three times with lysis buffer, boiled for 5 min in Laemmli sample buffer, and used for immunoblot analysis. As a control, protein A/G PLUS Agarose beads were incubated with monoclonal antibodies or cell lysates alone.

For the western blot analysis, obtained samples were separated by 10% SDS-PAGE and transferred onto PVDF membrane (Millipore, Billerica, MA). The non-specific binding was blocked with 5% skim milk in PBST (140 mM NaCl, 2.6 mM KCl, 10 mM Na _2_HPO _4_, 1.8 mM KH _2_PO _4_, 0,05% Tween-20, pH 7.4) for 1 h at RT, and then incubated with anti-TBR2/Eomes rabbit antibodies at 1:500 (Abcam Cat# ab23345, RRID:AB_778267) or anti-S6K1 C-terminal rabbit polyclonal antibodies 1:3000 overnight at 4°C. After washing three times with PBST, HRP-conjugated goat anti-rabbit IgG (Jackson ImmunoResearch Labs Cat# 111-035-144, RRID:AB_2307391) in 1:10 000 dilution were incubated with the membrane for 1 h at RT. The signal was developed using an enhanced chemiluminescence system (Fluco) and then exposed to Agfa X-ray film.

## Results and discussion

### Immunochemical detection of S6K1 subcellular localization in human breast cancer cells

**Figure 1.**
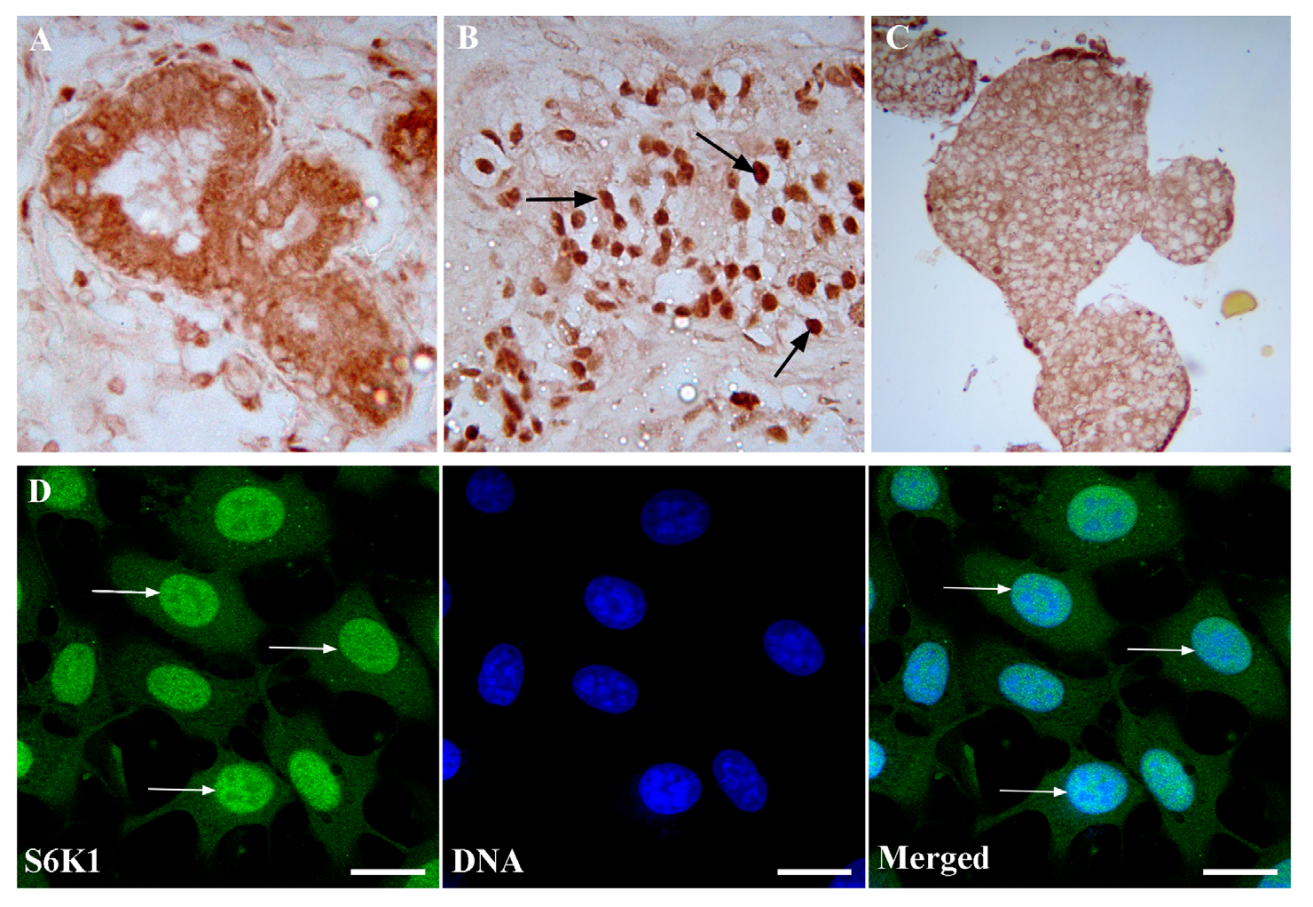
S6K1 subcellular localization in breast cancer cells *in vivo* and *in vitro*. ( **A**) Immunohistochemical detection of S6K1 in human conditionally normal breast tissue. Magnification 400x. ( **B**) Immunohistochemical analysis of subcellular distribution of S6K1 in human breast cancer tissue. Magnification 400x. Arrows indicate the staining in the nuclei of the cells. ( **C**) Immunohistochemical detection of S6K1 in fixed MCF-7 multicellular spheroids. Magnification 200x. ( **D**) Immunofluorescence image of S6K1 subcellular distribution (green) in 40% confluent MCF-7 cell monolayer. Arrows indicate the S6K1 localization in the nuclei of the cells. DNA was counterstained with DAPI (blue). Scale bars correspond to 20 µm. The data are representative of three independent experiments.

Firstly, the subcellular distribution of S6K1 was determined in the histological sections of human breast cancer and normal tissues. As in previous studies, we also observed the preferential nuclear localization of S6K1 in the malignant breast cells ( Bostner *et al*., 2015; Filonenko *et al*., 2004) and mainly cytoplasmic one in conditionally normal adjustment tissues ( Figure 1A, B).

In recent years, the 3D cell culture systems have been shown to provide numerous advantages to study tumor growth in a more physiologically relevant environment ( Bingel *et al*., 2017). This motivated us to further compare the intracellular distribution of S6K1 in MCF7 cells grown as either multicellular spheroids or a conventional monolayer cell culture. In the multicellular spheroids of MCF7 cells, we detected a strong accumulation of S6K1 in the cytoplasm and its reduction in the nuclei ( Figure 1C). In contrast to this, in the 40–60% confluent monolayer of MCF7 cells S6K1 was localized mostly to the nucleus, with the moderate signal in the cytoplasm of the same cells ( Figure 1D).

### Nucleocytoplasmic redistribution of S6K1 in MCF-7 cells at different cell density

**Figure 2.**
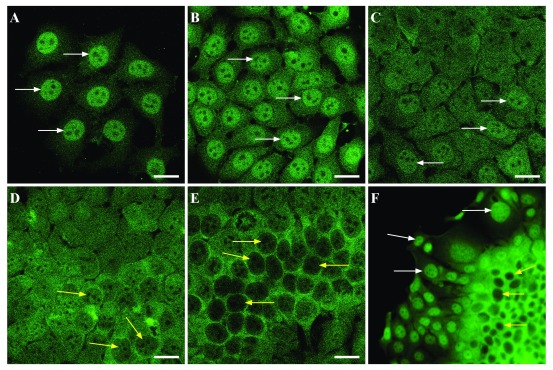
S6K1 relocalizes from the nucleus to the cytoplasm during formation of the confluent monolayer of MCF-7 cells. MCF-7 cells were seeded onto glass coverslips in the density 10 000 cells/well ( **A**), 30 000 cells/well ( **B**), 50 000 cells/well ( **C**), 70 000 cells/well ( **D**), 100 000 cells/well ( **E**), and cultivated for 48 hours. Then cells were fixed and stained with anti-S6K1 antibody (green). White arrows indicate the S6K1 localization in the nuclei of the cells, yellow arrows indicate the decreased staining in the nuclei. Scale bars are 20 µm. The images are representative of three independent experiments. ( **F**) MCF-7 cells were cultured to form a super-confluent monolayer. Then fragments of the monolayer were gently detached by short incubation with trypsin, transferred on the coverslip and left for 48 hours to grow. After this the fragments were fixed and stained with anti-S6K1 antibody (green). White arrows indicate the positive reaction in the nuclei of the cells at the leading edge of the monolayer fragment; yellow arrows indicate the decreased staining in the nuclei of the cells at the center of the monolayer fragment. Magnification 400x.

A significant difference in S6K1 localization in monolayer and spheroid cultures can be caused by differences in cell growth conditions in two different types of culture. Such differences could be potentially caused by a cascade of intracellular signaling events induced by cell-matrix adhesion or intercellular interactions. One may assume that the S6K1 could be involved in such intracellular rearrangement. To clarify this, we analyzed the S6K1 subcellular localization in MCF-7 cells cultured at different densities. The immunofluorescence analysis revealed the changes of S6K1 localization from the nucleus to the cytoplasm correlating with increased cell culture density ( Figure 2A–E). At the lowest cell density level, S6K1 was observed predominantly in the nuclei of cultured cells whereas at the highest cell density S6K1 was concentrated in the cytoplasm.

In order to further asses the possible connection between the subcellular localization of S6K1 and the cell density, we utilized the following approach. After reaching approximately 90% of confluence, the monolayer of MCF-7 cells was gently detached from growth surface by short treatment with trypsin (w/o EDTA), and placed in fresh culture medium. Subsequent cultivation of these monolayer fragments for additional 48 h led to still high cell density in the center of fragments and decreased density of cells at the edges of the fragments. Immunofluorescence analysis of this heterogenous population of the cells revealed that cell spreading at the edges of the dense fragments was accompanied by the alterations in S6K1 localization from predominantly cytoplasmic to nuclear ( Figure 2F). 

Unedited images that were used in Figure 1 and Figure 2, showing S6K1 subcellular localization in breast normal tissue, cancer tissue, and in MCF-7 cells monolayer Click here for additional data file. Copyright: © 2018 Kosach V et al. 2018 Data associated with the article are available under the terms of the Creative Commons Zero "No rights reserved" data waiver (CC0 1.0 Public domain dedication).

### Subcellular localization of S6K1 in migrating MCF-7 cells

**Figure 3.**
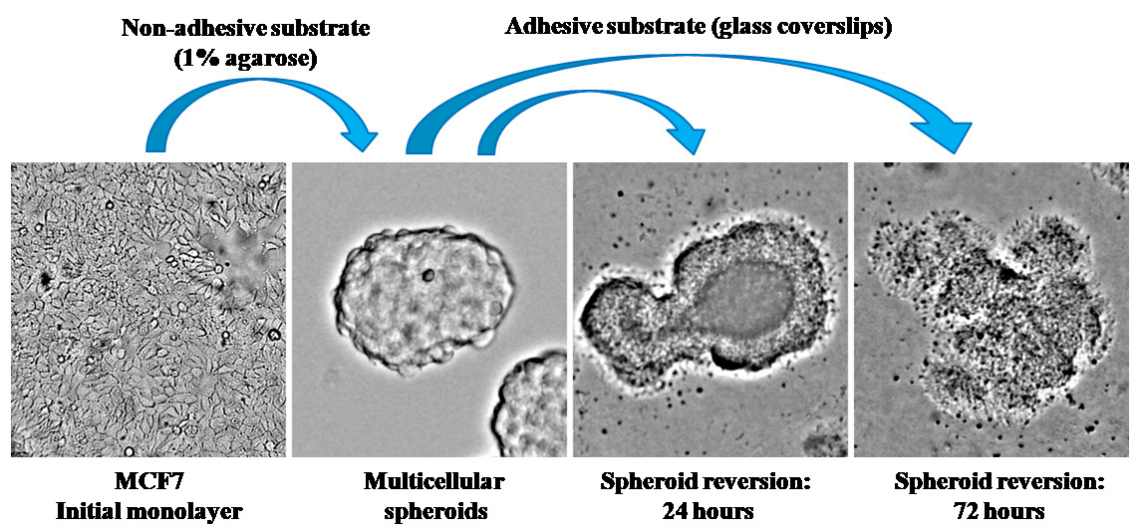
Scheme of the multicellular spheroid formation and initiation of cell migration. To generate multicellular spheroids, MCF-7 cells were seeded in the Petri dishes precoated with 1% agarose, and cultivated for 72 hours. To analyze cell migration, obtained spheroids were transferred onto glass coverslips and cultured for 24 or 72 hours. Transmitted light images of the spheroids general view were taken by Leica DM1000 (Leica, Germany). Magnification 200x.

**Figure 4.**
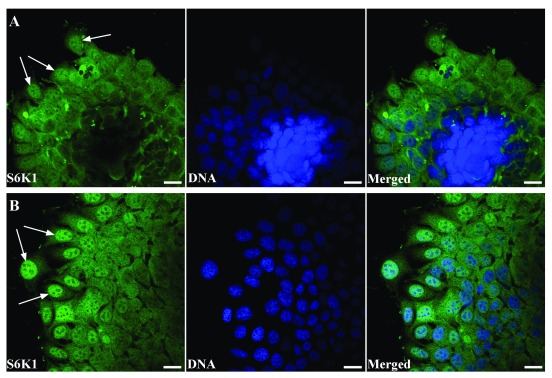
S6K1 shuttles to the nuclei during spheroid-to-monolayer reversion. ( **A**) Immunofluorescence analysis of S6K1 subcellular localization (green) in the MCF-7 spheroid reversed for 24 hours. Arrows indicate predominantly nuclear distribution of the S6K1 in the migrating cells. DNA was counterstained with DAPI (blue). Scale bars correspond to 20 µm. ( **B**) Immunofluorescence analysis of S6K1 subcellular localization (green) in the MCF-7 cells at the leading age of spheroid reversed for 72 hours. Arrows point to predominantly nuclear distribution of the S6K1 in the migrating cells. DNA was counterstained with DAPI (blue). Scale bars correspond to 20 µm. The images are representative of three independent experiments.

Obtained data led to the hypothesis that there is a possible relation between the initiation of cell migration and the relocalization of S6K1. Among the variety of cell migration models, the approaches based on the 3D cell cultures are the ones that provide several unique advantages for studying tumor cell migration and invasion ( Metzger *et al*., 2017). As mentioned previously, many studies describe the structural and physiological similarity of multicellular spheroid organization to the structure of solid malignant tumors ( Rodrigues *et al*., 2018). Also, the transformation of 3D multicellular spheroids into the 2D cell colonies upon contact with adhesive surface can be realized only through cell migration, in contrast to the monolayer migration assays, where cell proliferation also plays a role. Therefore, spheroid to monolayer reversion model to assess the nucleocytoplasmic distribution of S6K1 was utilized ( Figure 3). We applied the immunofluorescence analysis of cultured cells 24 and 72 hours after initiation of the MCF7 spheroid migration. Obtained data suggested that there is a significant relocalization of S6K1 from the cytoplasm into the nuclei in course of cell migration ( Figure 4A, B). The cells, remaining within the spheroid retained the positive cytoplasmic and negative nucleic reaction for S6K1, similar to the cells of spheroid at histological sections regardless of their remoteness from the edge of the spheroid ( Figure 1C), while the migrating cells at the edge of the spreading spheroid demonstrated strong accumulation of S6K1 in the nuclei ( Figure 4B).

Unedited images from Figure 3 Click here for additional data file. Copyright: © 2018 Kosach V et al. 2018 Data associated with the article are available under the terms of the Creative Commons Zero "No rights reserved" data waiver (CC0 1.0 Public domain dedication).

**Figure 5.**
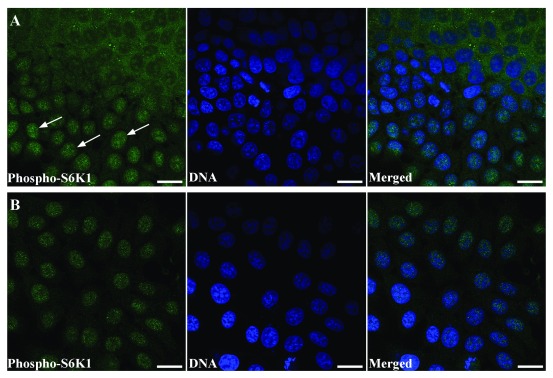
Immunofluorescence analysis of phospho-S6K1 (T389) subcellular localization. ( **A**) Confocal image of MCF-7 cells in spheroid-to-monolayer reversion model. Cells were stained with anti-phospho-S6K1 (T389) (green). DNA was counterstained with DAPI (blue). Scale bars correspond to 20 µm. Arrows indicate the staining in the nuclei of the migrating cells at the leading edge of the spheroid. ( **B**) Confocal image of monolayer culture of the MCF-7 cells stained with anti-phospho-S6K1 antibody (T389) (green). DNA was counterstained with DAPI (blue). Scale bars correspond to 20 µm. The images are representative of two independent experiments.

The mTOR dependent Thr389 phosphorylation of S6K1 is the most frequently used marker for the S6K1 activity ( Romanelli *et al*., 2002). Therefore, we analyzed the phosphorylation status of S6K1 in MCF-7 cells during spheroid transformation into monolayer by immunofluorescence analysis. Overall, the pattern of phospho-S6K1 distribution was similar to that observed for total S6K1 ( Figure 5). In particular, in the central part of the spheroid, S6K1 was mainly observed in the cytoplasm (however some of the nuclei were positive), whereas the cells at the leading edge of spheroid demonstrated predominant nuclear localization of phospho-S6K1 ( Figure 5A). Also, strong nuclear localization of phospho-S6K1 (Thr389) was revealed in monolayer culture of MCF-7 cells ( Figure 5B).

Unedited images that were used in Figure 4 and Figure 5, showing S6K1 and phospho-S6K1 (T389) subcellular localization during MCF-7 cell migration Click here for additional data file. Copyright: © 2018 Kosach V et al. 2018 Data associated with the article are available under the terms of the Creative Commons Zero "No rights reserved" data waiver (CC0 1.0 Public domain dedication).

### S6K1 function in nuclei and cell migration

While our data suggested that activation of cell locomotor function is accompanied by cytoplasm/nuclear shuttling of S6K1, the biological meaning of the event was not clear. One of the possible explanations could be the implication of S6K1 in the regulation of transcription factors affecting expression of genes that control cell migration.

That’s why, we analyzed the subcellular distribution of several transcription factors, which are known to be regulated by the mTOR/S6K signaling pathway and activated in migrating cells either in the cancer tissues or in the process of embryonic development. One of them is the mammalian transcription factor CDX2, which plays a key role in intestinal development and differentiation. It has been previously described that reduced expression of CDX2 may contribute to the colon tumorigenesis through involvement in the mTOR-mediated chromosomal instability ( Aoki *et al*., 2003). Fusion of another transcription factor ERG and androgen-responsive TMPRSS2 serine protease has been shown to contribute to the development of the prostate cancer. There is also a strong correlation between TMPRSS2-ERG fusion and activation of mTOR/S6K pathway ( Faraj *et al*., 2013; King *et al*., 2009). The third transcription factor chosen for this study was T-box transcription activator Eomesodermin (or TBR2) ( Conlon *et al*., 2001), which was also described as one of the targets for anticancer therapy. It has been shown that siRNA knockdown of Eomesodermin in human hepatocellular carcinoma could significantly decrease anchorage-independent cell growth ( Gao *et al*., 2014). Besides this, TBR2 has also been shown to be involved in the process of lymphocyte differentiation. The mTOR-dependent regulation of expression of transcription factors T-bet and Eomesodermin has been shown to be heavily involved in the determination of effector of memory cell fates in CD8+ T cells ( Cui *et al*., 2016).

**Figure 6.**
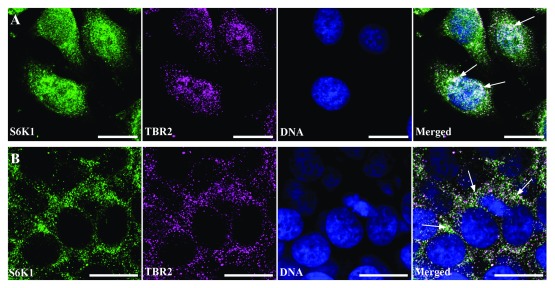
S6K1 partially colocalizes with transcription factor TBR2 in MCF-7 cells. ( **A**) Immunofluorescence image of low density monolayer culture of the MCF-7 cells co-stained with anti-S6K1 (green) and anti-TBR2 (magenta) antibodies. DNA was counterstained with DAPI (blue). Scale bars correspond to 20 µm. Arrows indicate the regions of S6K1 and TBR2 colocalization. ( **B**) Immunofluorescence image of high density monolayer culture of the MCF-7 cells double stained with anti-S6K1 (green) and anti-TBR2 (magenta) antibodies. DNA was counterstained with DAPI (blue). Scale bars correspond to 20 µm. Arrows point out to the regions of S6K1 and TBR2 colocalization. The images are representative of two independent experiments.

Our immunofluorescence analysis of subcellular distribution of S6K1 and mentioned transcription factors in MCF-7 cells revealed that ERG was present in scant quantities or not determined at all in MCF-7 cells ( Dataset 4; ( Kosach *et al*., 2018d)). CDX2 staining led to identification of positive dots predominantly in the nuclei of the MCF7 cells, however, CDX-2 and S6K1 colocalization was not detectable by confocal microscopy ( Dataset 4; ( Kosach *et al*., 2018d)). TBR2/Eomesodermin positive speckles were observed in the cytoplasm as well as in nuclei of the cells ( Figure 6A, B). In both cases, partial but intense colocalization of TBR2 and S6K1 was detected. Moreover, in the low-density monolayer, when S6K1 localized mainly in the cell nuclei, TBR2 was observed predominantly in the nuclei as well. In the high-density monolayer cultures, where S6K1 was distributed in the cell cytoplasm, TBR2 also displayed a similar pattern of intracellular distribution ( Figure 6A, B).

Unedited images of S6K1 colocalization with transcription factors TBR2 (Figure 6), ERG (Dako, Cat#M7314), and CDX2 (Abcam Cat# ab76541, RRID:AB_1523334) Click here for additional data file. Copyright: © 2018 Kosach V et al. 2018 Data associated with the article are available under the terms of the Creative Commons Zero "No rights reserved" data waiver (CC0 1.0 Public domain dedication).

For quantitative characterization of S6K1 and TBR2 colocalization, Pearson coefficient (Rr) and Manders coefficient (M1 and M2) analysis was performed on background-subtracted images using JACoP ImageJ plugin ( Bolte & Cordelières, 2006). M1 shown the colocalization of S6K1 with TBR2, whereas M2 expressed the pool of TBR2 colocalizing with S6K1. Colocalization analysis of S6K1 and TBR2 in low density monolayer revealed Pearson coefficient Rr= 0.55 +/- 0.113, M1= 0.999 +/-0.01, M2= 0.84 +/-0.087. To validate and describe the obtained degree of colocalization pre-defined image sets from Colocalization Benchmark Source were used. The closest benchmark was CBS007RGM that corresponded to 60% colocalization, thus indicating the medium level of colocalization between TBR2 and S6K1. A slightly lower but reliable colocalization of S6K1 and TBR2 was observed in a monolayer with a high density. Namely Pearson coefficient was Rr=0.47 +/- 0.064, Manders coefficients were M1=0.995 +/-0.004 and M2=0.62+/-0.187. So, a slightly higher level of S6K1 and TBR2 colocalization was revealed in MCF-7 cells grown in low density monolayer, when S6K1 and TBR2 localized mainly in the nuclei.

**Figure 7.**
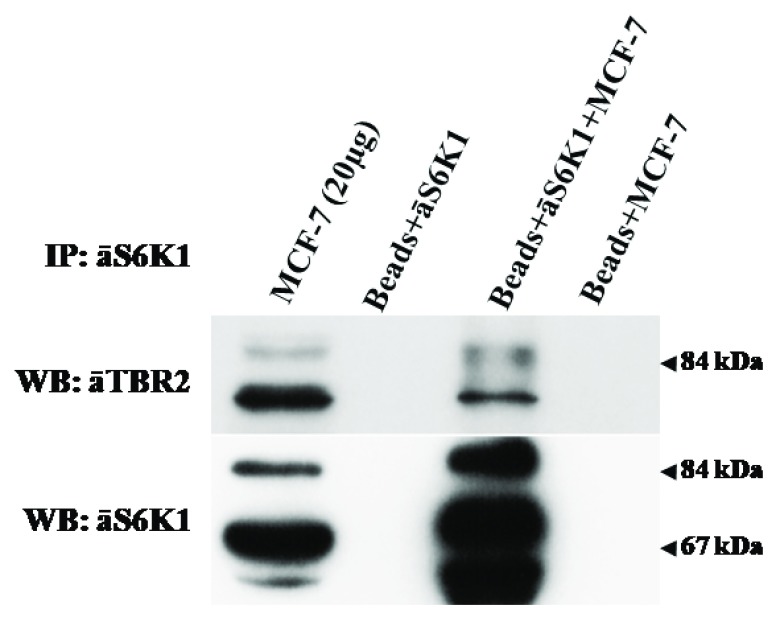
Co-Immunoprecipitation of S6K1 and TBR2 protein complex in the MCF-7 cells. Endogenous S6K1 was precipitated with anti-S6K1 mouse monoclonal antibodies immobilized on protein A/G PLUS Agarose beads (Santa Cruz Biotechnology). As a control, protein agarose beads were incubated with monoclonal antibodies or cell lysates alone. Immune complexes were analyzed by immunoblotting with anti-TBR2 rabbit antibodies (Abcam, ab23345) or anti-S6K1 C-terminal rabbit polyclonal antibodies. The data are representative of two independent experiments.

Further immunoprecipitation experiments also confirmed the physical interaction of S6K1 and TBR2 ( Figure 7). Protein complexes containing S6K1 were extracted from cultured MCF-7 cell lysate using anti-S6K1 antibodies and then blotted with antibodies to TBR2. Obtained results revealed the protein complex formation of S6K1 and TBR2, leading to the hypothesis of the possibility of TBR2 regulation via S6K1 mediated phosphorylation.

Unedited western blot images of co-immunoprecipitation of S6K1 and TBR2 used in Figure 7 Click here for additional data file. Copyright: © 2018 Kosach V et al. 2018 Data associated with the article are available under the terms of the Creative Commons Zero "No rights reserved" data waiver (CC0 1.0 Public domain dedication).

**Figure 8.**
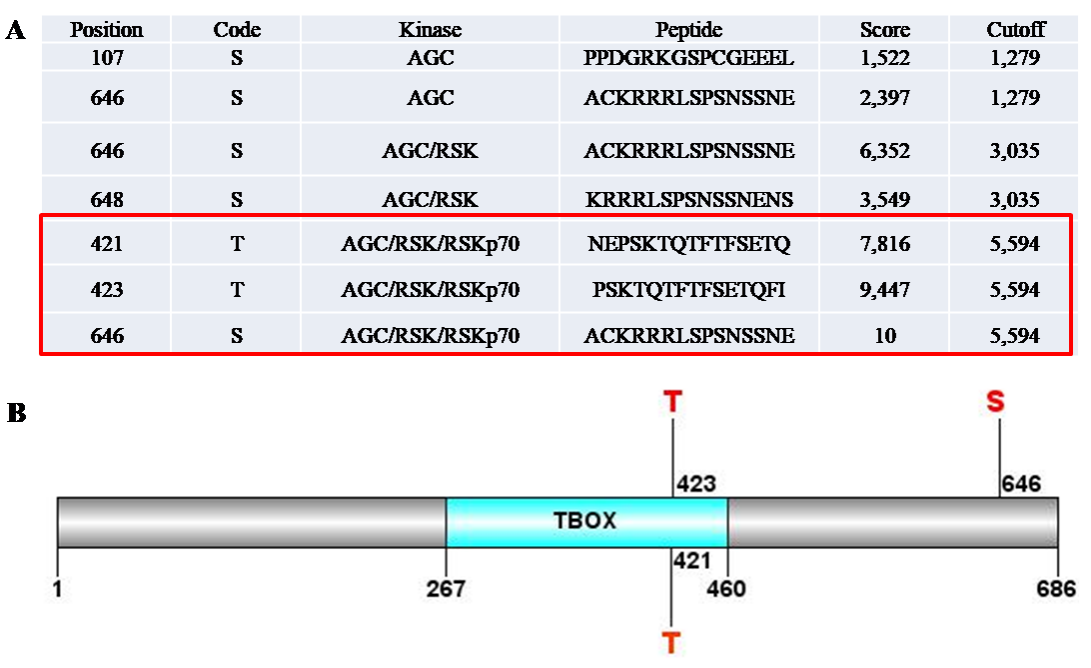
S6K1 possibly phosphorylates TBR2 at several residues. Group-based Prediction System v2.1 was used for bioinformatics analysis. It revealed that TBR2 contained three sites that could be phosphorylated by S6K1 with a high probability ( **A**). Two of them, Thr421 and Thr423, are located in the DNA binding domain of the TBR2. Third site Ser646 is located within the transcription activation domain at C-terminus of TBR2 ( **B**).

To assess this possibility of S6K1-mediated TBR2 phosphorylation, we performed a computational prediction of phosphorylation sites in TBR2 (GPS 2.1), which indicated several potential phosphorylation sites, and three of them (Thr421, Thr423, Ser646) could be phosphorylated by S6K1 with a high probability score ( Figure 8). Interestingly, both Thr421 and Thr423 were located in the DNA binding domain indicating that their phosphorylation could be related to the binding affinity of this transcription factor to the DNA. Another phosphorylation site (Ser646) was located within transcription activation domain at C-terminus of TBR2, which is thought to be involved in transcription activation. Taken together, this data suggests that S6K1 can be involved in the regulation of TBR2 transcription activity. However, further research is needed to confirm if S6K1 phosphorylates TBR2 *in vitro* and *in vivo*.

In the course of embryonic and postnatal development, Eomesodermin has been shown to induce the expression of a large spectrum of mesodermal genes in all types of mesodermal cells, which could also be expressed in malignant cells of non-mesodermal origin ( Reim *et al*., 2017; Russ *et al*., 2000).

Considering the multiplicity of S6K1 substrates, possible phosphorylation of the TBR2 transcription factor is not the only reason for the movement of the kinase from the cytoplasm into the nucleus of migrating cells. However, the proposed interaction can partially explain the accumulation of kinase in the nucleus of moving cells. In addition to the previously known classical nuclear substrates of S6K1, in case of breast cancer, it is necessary to note that this kinase can activate estrogen receptor-α, which is a nuclear transcription factor by its phosphorylation at Ser167 in a ligand-independent manner ( Yamnik & Holz, 2010). Besides, recent data indicate that S6K1 is targeted by histone acetyltransferases p300 and p300/CBP-associated factor (PCAF). The significance of this acetylation is not fully clear, but by analogy with S6K2, it is assumed that S6K1 is involved in the regulation of the transcription process ( Fenton *et al*., 2010). Summing up, there are a number of data confirming the nuclear localization of S6K1, but the role that S6K1 performs in the nucleus of migrating malignant cells require further investigation.

## Conclusions

For the first time, this study revealed the interconnection between MCF-7 cell density and S6K1 subcellular distribution: nuclear localization of the kinase was observed at low density monolayer, while in the confluent monolayer S6K1 was detected predominantly in the cytoplasm. Besides, S6K1 nucleocytoplasmic relocalization was revealed in migrating MCF-7 cells using spheroid-to-monolayer reversion model.

## Data availability

The data referenced by this article are under copyright with the following copyright statement: Copyright: © 2018 Kosach V et al.

Data associated with the article are available under the terms of the Creative Commons Zero "No rights reserved" data waiver (CC0 1.0 Public domain dedication). 

F1000Research: Dataset 1. Unedited images that were used in Figure 1 and Figure 2, showing S6K1 subcellular localization in breast normal tissue, cancer tissue, and in MCF-7 cells monolayer. 10.5256/f1000research.15447.d214430 ( Kosach *et al*., 2018a).

F1000Research: Dataset 2. Unedited images from Figure 3, 10.5256/f1000research.15447.d214431 ( Kosach *et al*., 2018b).

F1000Research: Dataset 3. Unedited images that were used in Figure 4 and Figure 5, showing S6K1 and phospho-S6K1 (T389) subcellular localization during MCF-7 cell migration. 10.5256/f1000research.15447.d214432 ( Kosach *et al*., 2018c).

F1000Research: Dataset 4. Unedited images of S6K1 colocalization with transcription factors TBR2 ( Figure 6), ERG, and CDX2. 10.5256/f1000research.15447.d214433 ( Kosach *et al*., 2018d).

F1000Research: Dataset 5. Unedited western blot images of co-immunoprecipitation of S6K1 and TBR2 used in Figure 7. 10.5256/f1000research.15447.d214434 ( Kosach *et al*., 2018e).
