# Supplementary material for: Nucleocytoplasmic distribution of S6K1 depends on the density and motility of MCF-7 cells in vitro
Source: F1000Res. 2018 Dec 17;7:1332. Originally published 2018 Aug 23. [Version 2] doi: 10.12688/f1000research.15447.2 (PMC6343231; doi:10.12688/f1000research.15447.2)
Supplement: Peer review history

# Peer review: Referee response for version 2

**DOI:** 10.5256/f1000research.18161.r42025

**Published:** 2019-01-18

**Authors:** Yegor Vassetzky

**Affiliations:** 1 French National Centre for Scientific Research(CNRS), UMR 8126, Institut Gustave Roussy (IGR), University of Paris-Sud (University of Paris XI), Villejuif, France

The authors have successfully replied to the criticism of this reviewer.

I have read this submission. I believe that I have an appropriate level of expertise to confirm that it is of an acceptable scientific standard.

# Peer review: Referee response for version 1

**DOI:** 10.5256/f1000research.16834.r38191

**Published:** 2018-10-05

**Authors:** Yegor Vassetzky, Svetlana Dokudovskaya

**Affiliations:** 1 French National Centre for Scientific Research(CNRS), UMR 8126, Institut Gustave Roussy (IGR), University of Paris-Sud (University of Paris XI), Villejuif, France; 2 French National Centre for Scientific Research(CNRS), UMR 8126, Institut Gustave Roussy (IGR), University of Paris-Sud (University of Paris XI), Villejuif, France

The MS by Kosach et al. describes an interesting phenomenon, nucleocytoplasmic shuttling of S6K1 in MCF-7 cells. The authors established a relationship between MCF-7 monolayer density and subcellular localization of the kinase. The work is worthy of publication, but the MS should be significantly shortened as is contains some redundant information and speculations. Proofreading by a native English-speaker is also recommended.

 Major point:

 The putative phosphorylation of TBR2 by S6K1 is too speculative and not convincing to use it as one of the major conclusions of the papers. It should be either confirmed experimentally or removed from the abstract and the main results of the paper.  

 Minor points:

 Material and Methods:

 p4 Please remove “if you are interested in obtaining this antibody, please contact the corresponding author“;

 p4 The anti-ERG and anti-CDX antibodies are not used in the main Figures. Please move information about these antibodies form the Materials and Methods section to legends of the supplementary figures;

 Results and discussion:

 P5 Please remove the first paragraph or move it to the introduction section.

 P5 “For the first step of the present study” is redundant

 Figure 1,5 legend. Please replace “positive reaction” with “staining”

 P6  The following phrase is unclear: “Since cell spreading can be considered as a stage of migration, the previous data led to the hypothesis…”

 Figure 6. It is unclear whether the images represent confocal sections or reconstituted 3D images. Colocalization of S6K1 and TBR2 should be analyzed quantitatively, eg using ImageJ tools for colocalization analysis.  It is also unclear whether TBR2 shuttles between the nucleus and the cytoplasm similarly to S6K1.

 P9. S6K1 is a serine threonine kinase and the putative phosphorylation of TBR2 by S6K1 should concern threonines rather than tyrosines. I also propose to remove the bioinformatic part and the Figure 8 as they are too speculative. Indeed, many other kinases may potentially phosphorylate TBR2 basing on the bioinformatic analysis, and the authors did not even show whether TBR2 is indeed phosphorylated.

 Figure 7. TBR2 has a molecular weight of 84 kDa of TBR2, but the major band has a lower molecular weight. This should be explained in the Results section.

We have read this submission. We believe that we have an appropriate level of expertise to confirm that it is of an acceptable scientific standard, however we have significant reservations, as outlined above.

# Response

**Published:** 2018-12-03

**Authors:** Viktoriia Kosach

**Affiliations:** Institute of Molecular Biology and Genetics, NAS of Ukraine, Ukraine

We thank the reviewers for deep and knowledgeable revision of this manuscript. Please, find below our response to the reviewers’ comments point by point.

1. *Major point:The putative phosphorylation of TBR2 by S6K1 is too speculative and not convincing to use it as one of the major conclusions of the papers. It should be either confirmed experimentally or removed from the abstract and the main results of the paper.*

We have withdrawn the mentioned statements from the conclusions and the abstract, and left as a hypothesis in the text of our article. 

         2. Minor points

*p4 Please* *remove “if* *you* *are* *interested* *in* *obtaining* *this* *antibody, please* *contact* *the* *corresponding* *author“;*

 We could not remove it, as this sentence is a recommendation of the F1000Research Editorial board, and it informs the other researchers, where they could obtain these antibodies to replicate the study.

*p4* *The anti-ERG and anti-CDX antibodies are not used in the main Figures. Please move information about these antibodies form the Materials and Methods section to legends of the supplementary figures;*

 We agree. We have moved mentioned information to the legend of Dataset 4.

*Results and* *discussion:*

*P5 Please* *remove* *the* *first* *paragraph* *or* *move* *it* *to* *the* *introduction* *section.*

*P5 “For* *the* *first* *step* *of* *the* *present* *study” is* *redundant*

*Figure 1,5 legend. Please* *replace “positive* *reaction” with “staining”*

*P6 The* *following* *phrase* *is* *unclear: “Since* *cell* *spreading* *can* *be* *considered* *as a stage* *of* *migration, the* *previous* *data* *led* *to* *the* *hypothesis…”*

 We agree with all comments. We have corrected them in version 2 according to reviewers’ queries.

*Figure 6. It* *is* *unclear* *whether* *the* *images* *represent* *confocal* *sections* *or* *reconstituted 3D images. Colocalization* *of S6K1 and TBR2 should* *be* *analyzed* *quantitatively, eg* *using* *ImageJ* *tools* *for* *colocalization* *analysis.  It* *is* *also* *unclear* *whether TBR2 shuttles* *between* *the* *nucleus* *and* *the* *cytoplasm* *similarly* *to S6K1.*

 The images represent confocal sections. Colocalization of S6K1 and TBR2 was analysed using ImageJ JaCoP plugin, as it is indicated in article. As well as S6K1, we observed TBR2 nucleo/cytoplasmic relocalization that depended on cell density. Moreover, TBR2 shuttled between cytoplasm and nuclei similarly to S6K1 during spheroid to monolayer reversion. But quantitatively the level of colocalization of S6K1 and TBR2 we detected on MCF-7 monolayer at different density, because the cells are in more similar condition reliable for image analysis than in course of 3D spheroid transformation into 2D monolayer colony.

*P9. S6K1 is a serine* *threonine* *kinase* *and* *the* *putative* *phosphorylation* *of TBR2 by S6K1 should* *concern* *threonines* *rather* *than* *tyrosines. I also* *propose* *to* *remove* *the* *bioinformatic* *part* *and* *the* *Figure 8 as* *they* *are* *too* *speculative. Indeed, many* *other* *kinases* *may* *potentially* *phosphorylate TBR2 basing* *on* *the* *bioinformatic* *analysis, and* *the* *authors* *did* *not* *even* *show* *whether TBR2 is* *indeed* *phosphorylated.*

 We agree that there was erratum concerning tyrosin and threonine. We have fixed it.

 As co-immunoprecipitation revealed the existence of S6K1-TBR2 protein complex in MCF-7 cells, we considered the theoretical probability of TBR2 phosphorylation by S6K1 kinase. Of course, this does not exclude the presence of other effectors of this transcription factor, but gives us the prerequisites for hypothesis. We removed this statement from the conclusions of the article.

*Figure 7. TBR2 has a molecular weight of 84 kDa of TBR2, but the major band has a lower molecular weight. This should be explained in the Results section.*

*​​​​​​​*

 The antibody supplier also state that anti-TBR2 antibodies detects two bands in immunoblot analysis. We have found information that 4 splice isoforms of TBR2 with lower molecular weight are known today (https://www.uniprot.org/uniprot/O95936).

# Peer review: Referee response for version 1

**DOI:** 10.5256/f1000research.16834.r37832

**Published:** 2018-09-28

**Authors:** Olivier E. Pardo

**Affiliations:** 1 Division of Cancer, Department of Surgery and Cancer, Imperial College London, London, UK

The study by Kosach et al. clearly shows for the first time differential nucleo-cytoplasmic localisation of S6K1 in response to cell density and migration. It also provides some information about the type of role that S6K1 may play in the nucleus and the biological processes that may be associated.

 The results shown are convincing and the quality of the data is sufficient to support the conclusions of the authors. Therefore, it is the opinion of this reviewer that the present manuscript should be accepted. However, the authors may want to address the comments below so as to improve the quality/impact of the manuscript:

  

1. At the end of the fourth paragraph of the Introduction, the authors refer to EGF as “endothelial growth factor”. This should be corrected to “epithelial growth factor”.
2. The value of the first paragraph of the Results section (There are several... of different function.”) is not clear and should be deleted.
3. On Page 13 of the manuscript, the authors refer to 2 residues on TRB2, Tyr421 and Tyr423, as potential sites phosphorylated by S6K1. S6K2 is a Ser/Thr kinase and therefore unable to phosphorylate Tyr residues. Are the authors referring to Thr sites instead? Please clarify.
4. The authors argue that interaction of S6K1 with TRB2 may be involved in the migration of cells out of microspheres onto 2D layers. This is an interesting proposition and the manuscript would benefit from an experiment being performed using siRNAs to TRB2 and showing that this impacts either S6K1 distribution or the movement of cells out of the microspheres.
5. The style of the paper is poor in places and could be improved prior to indexing.

I have read this submission. I believe that I have an appropriate level of expertise to confirm that it is of an acceptable scientific standard.

# Response

**Published:** 2018-12-03

**Authors:** Viktoriia Kosach

**Affiliations:** Institute of Molecular Biology and Genetics, NAS of Ukraine, Ukraine

We thank the reviewer for expert opinion and insightful comments on our study. We appreciate these comments, and we have tried to answer step by step. Please, find below our response to the reviewers’comments.

1. *At the end of the fourth paragraph of the Introduction, the authors refer to EGF as “endothelial growth factor”. This should be corrected to “epithelial growth factor”.*

We agree. It was erratum concerning EGF, and we have fixed it.

1. *The value of the first paragraph of the Results section (There are several... of different function.”) is not clear and should be deleted.*

We have deleted mentioned paragraph.

1. *On Page 13 of the manuscript, the authors refer to 2 residues on TRB2, Tyr421 and Tyr423, as potential sites phosphorylated by S6K1. S6K1 is a Ser/Thr kinase and therefore unable to phosphorylate Tyr residues. Are the authors referring to Thr sites instead? Please clarify.*

Yes, of course, S6K1 is Ser/Thr protein kinase, and we wrote about Threonine 421 and Threonine 423 residues on TRB2, but, unfortunately, there was erratum in the text. We have fixed it in version 2.

1. *The authors argue that interaction of S6K1 with TRB2 may be involved in the migration of cells out of microspheres onto 2D layers. This is an interesting proposition and the manuscript would benefit from an experiment being performed using siRNAs to TRB2 and showing that this impacts either S6K1 distribution or the movement of cells out of the microspheres*.

This is a very interesting suggestion. Also, to our mind, interaction of S6K1 and TBR2 could be just one from the possible reasons of S6K1 subcellular redistribution in course of MCF-7 cell migration. The level of their colocalization is statistically significant, but not very high, so, under TBR2 down-regulation we can obtain partial alteration of S6K1 relocalization that will be difficult to assay. Therefore, in the present work, we so far only point to the revealed colocalization. And we hope to develop this area of research in future.

1. *The style of the paper is poor in places and could be improved prior to indexing.*

The manuscript was proofread by a person with advanced level of scientific English. We believe that this greatly improved the style of the text.
